# Scaling-up filariasis lymphoedema management into the primary health care system in Kerala State, Southern India: a case study in healthcare equity

**DOI:** 10.1186/s40249-022-00936-6

**Published:** 2022-01-18

**Authors:** Suma T. Krishnasastry, Charles D. Mackenzie, Rajeev Sadanandan

**Affiliations:** 1 Filariasis Research Unit, WHO Collaborating Center for LF MMDP, Lymphatic Filariasis Morbidity Management and Disability Prevention, Department of Internal Medicine, Govt. T. D. Medical College Hospital, Kerala University of Health Sciences, Alappuzha, 688005 India; 2 NTD Support Center, Task Force for Global Health, Atlanta, GA 30030 USA; 3 Health Systems Transformation Platform, New Delhi, 110070 India

**Keywords:** Lymphatic filariasis, Health equity, India, Lymphoedema, Global program to eliminate lymphatic filariasis

## Abstract

**Background:**

Lymphatic filariasis (LF) remains one of the world’s most debilitating parasitic infections and is a major contributor to poor health in many endemic countries. The provision of continuing care for all those affected by LF and its consequences is an important component of the United Nations’ Sustainable Development Goals. The aim of this study is to integrate lymphedema care into the primary health care system of the State by developing lymphedema clinics at each district, through training of health personnel to fulfill WHO recommendation for morbidity management and disability prevention.

**Methods:**

Selected health care providers from all the districts in Kerala State of India participated in intensive training sessions endorsed by the State’s health administration. The six training sessions (from 5 June 2017 to 25 May 2018) included appropriate self-care information and development of individual plans for each participating institution to provide instruction and care for their lymphoedema patients. The learning achieved by attendees was assessed by pre- and post-training tests. The number of lymphoedema patients receiving care and instruction from the post-training activities of each participating institution was assessed from local records, 6 months after the conclusion of the training sessions.

**Results:**

One hundred and eighty-four medical personnel (91 doctors and 93 nurses) from 82 medical institutions were trained which quickly led to the establishment of active lymphoedema clinics providing the essential package of care (EPC) for lymphoedema patients at all the participating institutions. Six months after the training sessions the number of previously unidentified lymphoedema patients registered and receiving care at these clinics ranged from 296 to almost 400 per clinic, with a total of 3,477 new patients receiving training in EPC.

**Conclusions:**

Generalist health personnel, when appropriately trained, can provide quality lymphoedema care in public health settings and patients when provided services close to their home, are willing to access them. This is a feasible strategy for integrating long term care for LF patients into the national health system, and is a clear example of moving towards equity in health care for the medically underserved, and thus successfully addresses a major goal of the global program to eliminate lymphatic filariasis.

**Graphical Abstract:**

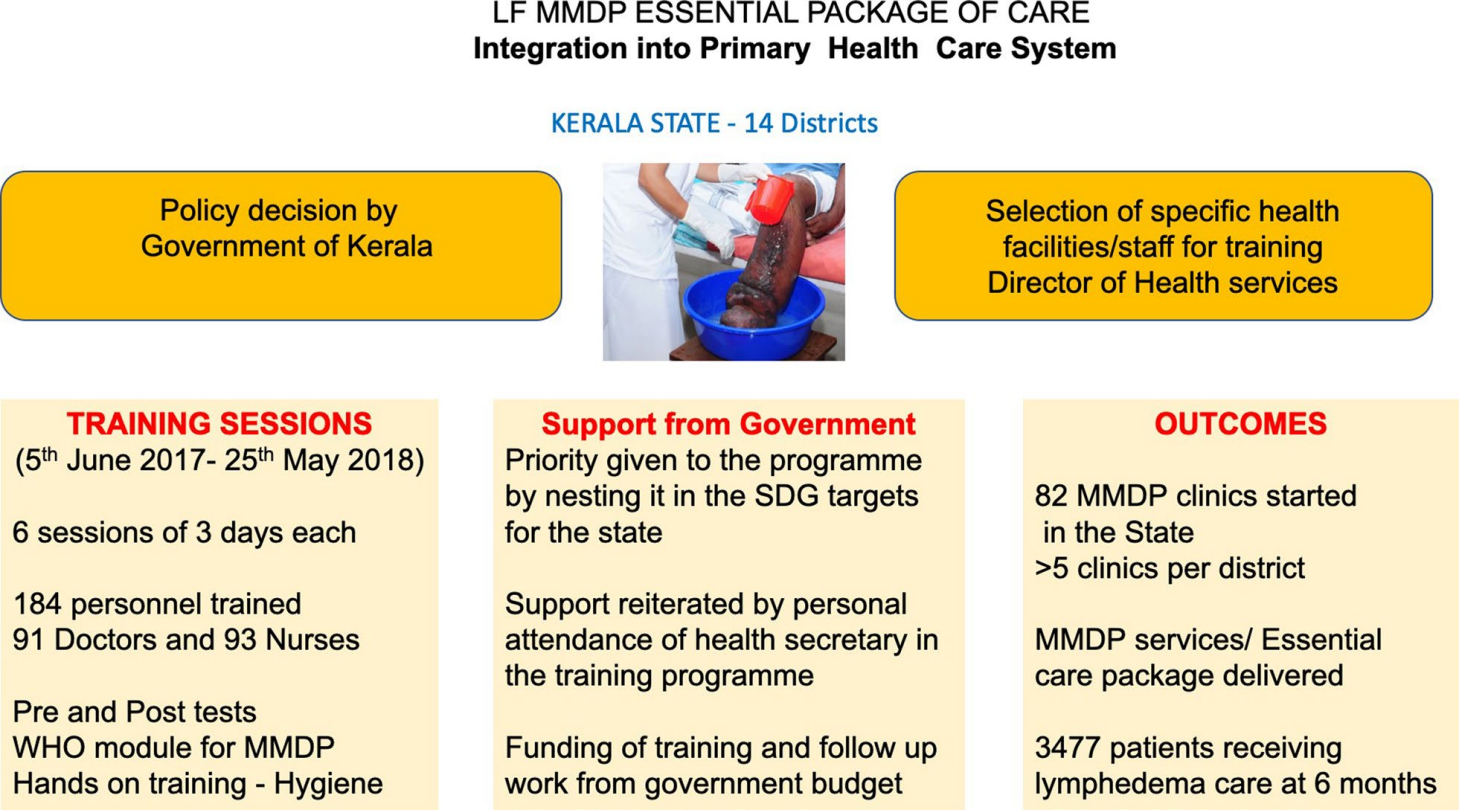

**Supplementary Information:**

The online version contains supplementary material available at 10.1186/s40249-022-00936-6.

## Background

Lymphatic filariasis (LF) remains one of the world’s most debilitating parasitic infections and is a major contributor to poor health as the second most common global cause of physical disability*.* It is estimated that there are 450 million infected and affected people in India, accounting for 40% of the global human LF infections, with a further estimated 450 million population still ‘at risk’of infection [1–4]*.* In 2000, in India around 7.44 million were suffering from LF-induced lymphoedema and approximately 12.88 million were suffering from hydrocele [5]. In 1997 the World Health Assembly targeted LF for global elimination of infection as a public health problem [6]. This initiative includes alleviating the suffering in those who already have the disease through Morbidity Management and Disability Prevention (MMDP). In addition to providing an “essential package of care” (EPC) to those suffering from LF [7, 8], there is the important additional recommendation for the integration of this medical support into the public health system of the endemic country. Such a goal is also in line with the global goals set out by the United Nations’ Sustainable Develop Goals (SDG) [9]. Such integration has often been a challenge for many LF endemic countries but has been an important goal for the filariasis program in highly endemic Kerala State, Southern India.

The major aim in the integration efforts in Kerala was to ensure the availability of the WHO recommended EPC for LF patients in all areas of the State, with the aim of providing 100% geographical coverage of the EPC for LF patients, and that at least one health facility designated for MMDP services per global program to eliminate lymphatic filariasis (GPELF) implementation unit (IU). In addition, as advised by WHO, these services should be provided at the appropriate level of the government health system, and be of good quality. This current communication describes the activities carried to fulfill this integration efforts and the successes achieved in addressing this important health initiative in this filariasis endemic region of India.

## Methods

### Study population

Kerala State in south India has a population of approximately 34.7 million people and 11/14 districts are known to be endemic for lymphatic filariasis (all districts except Pathanamthitta, Idukki and Wyanad). Although GPELF was officially launched by WHO in 2000, MDA was first started in Alappuzha, Kozhikode and Kannur districts in 1997. Subsequently, the MDA activities were extended to all 11 endemic districts with diethylcarbamazine and albendazole being given in a total of eight annual rounds of MDA, the last being carried out in 2012. Currently MDA is continuing only in certain areas of one district (Malappuram) where the infection has persisted.

### Training procedures

The Director of Health Services for Kerala State selected specific health facilities in every district (i.e., 14 districts), which included the three non-endemic districts as patients with LF clinical disease have been reported to reside in these districts; doctors and nurses from these health facilities were then selected for the training. The training was given to a total of 184 health care providers, in 6 sessions of 3 days each, starting on 5 June 2017 till 25 May 2018. The training was carried out by the staff of the Filariasis Research Unit at the Government TD Medical College Hospital, Alappuzha, Kerala. The number of health care providers trained and the dates of the training events are given in Table 1.

**Table 1:** Attendees at the lymphatic filariasis MMDP training sessions held in Kerala State, India (2017–2018)

Session	Training session dates	Duration of training session (days)	Number of doctors present	Number for staff nurses present	Total medical personnel present
1	5–8 June, 2017	3	16	17	33
2	21–23 August, 2017	3	13	15	28
3	29–31 August, 2017	3	15	15	30
4	7–9 March, 2018	3	15	15	30
5	22–24 March, 2018	3	17	17	34
6	23–25 May, 2018	3	15	14	29
Total participants			91	93	184

*MMDP* morbidity management and disability prevention

The training content followed the WHO Certified Training Module which encompasses components detailing the general background, as well as clinical and programmatic, aspects of LF. The learning objectives of the training sessions for the attendees were to: (1) to understand the background, the requirements and current status of GPELF; (2) to understand LF clinical disease focusing on lymphoedema, hydrocele, and acute attacks [adenolymphangitis (ADL)]; also to be aware of the recommended essential package of care; and to (3) learn details concerning the implementation of GPELF including situation analysis, selection and development of health facility to impart MMDP services, the documentation and reporting of services provided, and assessment of quality of services given by the health facility.

**Fig. 1:**
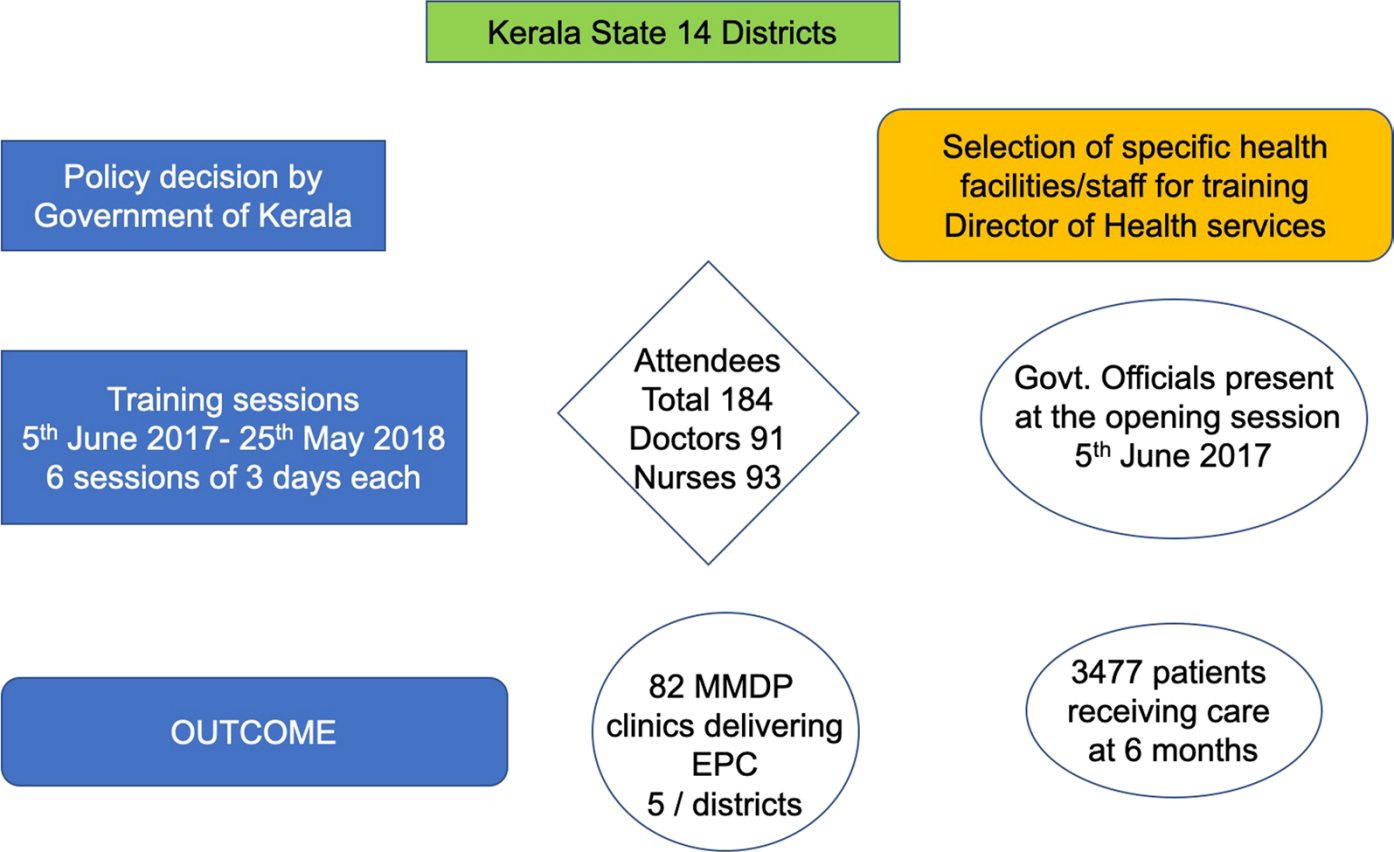
Flow diagram of procedures, major activities and outcomes activities in the Kerala Program

**Fig. 2:**
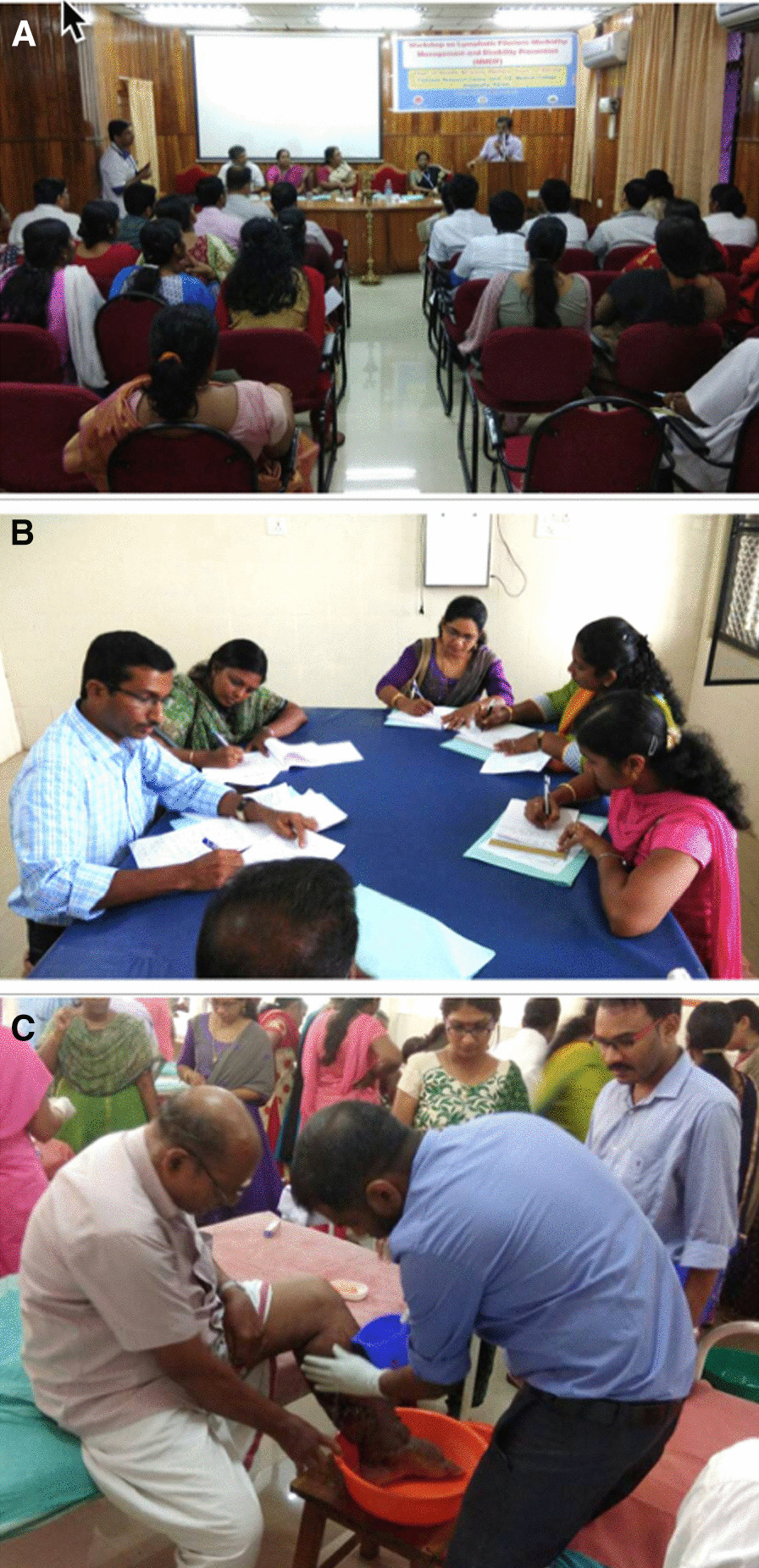
Training of medical staff in lymphoedema care. **A** Opening of training session. **B** Staff developing lymphoedema care plans for their individual institutions. **C** Direct hands-on experience for the medical staff in providing care to lymphoedema affected individuals

The longstanding WHO Filariasis Research Center in the Government TD Medical College of Alappuzha with considerable past experience in training, worked with Kerala’s health Administration to develop a comprehensive solid, state wide, system of specialized care for LF lymphoedema patients. The training of the medical staff occurred through organized, structured sessions over 6 months and included information, hands-on demonstrations and the development of specific LF lymphoedema care plans for the trainees’ hospitals (Figs. 1, 2).

### Assessment of activities and outcome

Each training session included interactive sessions, photo quizzes, as well as direct interaction with lymphoedema patients. Participants were instructed in taking clinical history, carrying out a physical examination, and given hands-on-training on limb hygiene and other details of lymphedema management. The participants were divided into groups according to their institutions and each group given the task of developing a proposal for initiating MMDP services at their own hospitals. The institutional plans that each group of participants developed during the training sessions were assessed for quality and content.

At the beginning of each of the six training sessions every attendee was administered a twenty-item questionnaire with questions covering both programmatic and clinical elements. The same questions were asked again at the conclusion of each training session. A few attendees (an overall total of 8 across all sessions) did not complete both tests. Throughout the training period regular conversations were held with the participants to obtain their opinions of the training and on any challenge, they might have had in implementing the instructions and advice they received.

The number of medical facilities that instituted a lymphoedema care system as result of attending the training sessions was assessed 1 year after the end of training sessions. The number of patients who received instruction in the package of self-care from these clinics at 6 months after the training session was also determined by survey of all participating institutions.

## Results

### Training sessions

One hundred and eighty-four medical personnel (91 doctors and 93 staff nurses) from 82 medical institutions were trained which led to the immediate establishment of lymphoedema clinics at all these institutions to provide the EPC for these patients (Tables 1, 2). Interviews with all the participants in all the six different sessions found that they were very enthusiastic about the training sessions and the included activities; participants were especially satisfied with the development, as part of the training program, of quality institutional proposals for starting and carrying out MMDP services for their own patients suffering from LF.

**Table 2:** The number of lymphatic filariasis (MMDP-active) clinics started in Kerala State, India (as reported mid-2018)

District	Number of staff trained	Number of institutions receiving training	Number of MMDP clinics started
Thiruvananthapuram	11	6	6
Kollam	14	7	7
Pathanamthitta	6	4	4
Alappuzha	20	6	10
Kottayam	6	3	3
Idukki	6	3	3
Ernakulam	14	7	7
Thrissur	13	5	3
Palakkad	28	13	5
Malappuram	21	9	11
Kozhikode	18	10	9
Wayanad	2	1	1
Kannur	12	4	6
Kasaragod	13	4	7
Total	184	82	82

*MMDP* morbidity management and disability prevention

### Feed back on the effectiveness of sessions

The structured questionnaire asked of the 144 attendees included questions related to the instructors/facilitators, to the organisation, and as well the opportunity to suggest changes/improvements in the format of the training. Comments about the instructors were all positive and no negative comments were made; those regarding the organisation were almost exclusively positive with an only a few minor comments related to logistic issues (e.g., “dinner should be provided”); a range of useful comments were made regarding improving the training session (e.g., providing certificates, involving the participants more actively, better infrastructural facilities). In summary, the vast majority of comments responding to the post-training interviews underscored the positive nature of the training sessions.

### Development of proposals for starting lymphedema clinics

A key component of the training sessions was the development of specific proposal for training and patient care in the respective home bases of the participants. These proposals included appraising their superintendents and district medical officers (DMOs) about the need for initiating the MMDP services, the training of the other health care providers in their own hospitals, carrying out infrastructure modifications, procuring the necessary materials for management of lymphedema and acute attacks, i.e., antibiotics and other drugs, antiseptic, anti-fungal ointments, and the acquisition of the necessary materials for limb hygiene measures. These plans also included introduction of appropriate information, education and communication (IEC) activities and maintenance of records and documents, as well planning for regular documentation and reporting on their ongoing activities.

### Support from State Government medical administration

An important factor in the success of these training sessions was the support from the State Health Administration. A policy decision was taken by the government that Kerala state should achieve LF elimination and MMDP in addition to MDA, was to be a key component of that strategy. To provide this in all parts of the State a team of health care providers—a doctor and staff nurse—from each Taluk Head Quarters hospitals should be given training in LF MMDP. These teams would then train others in their respective facilities, and thus ensure a wide distribution of the needed services across the state. The second example of important administrative support for the training activities was the attendance of high-level government officials at the opening of each session. This underscored the importance placed by the State Government in these training sessions.

### Assessment of learning

**Fig. 3:**
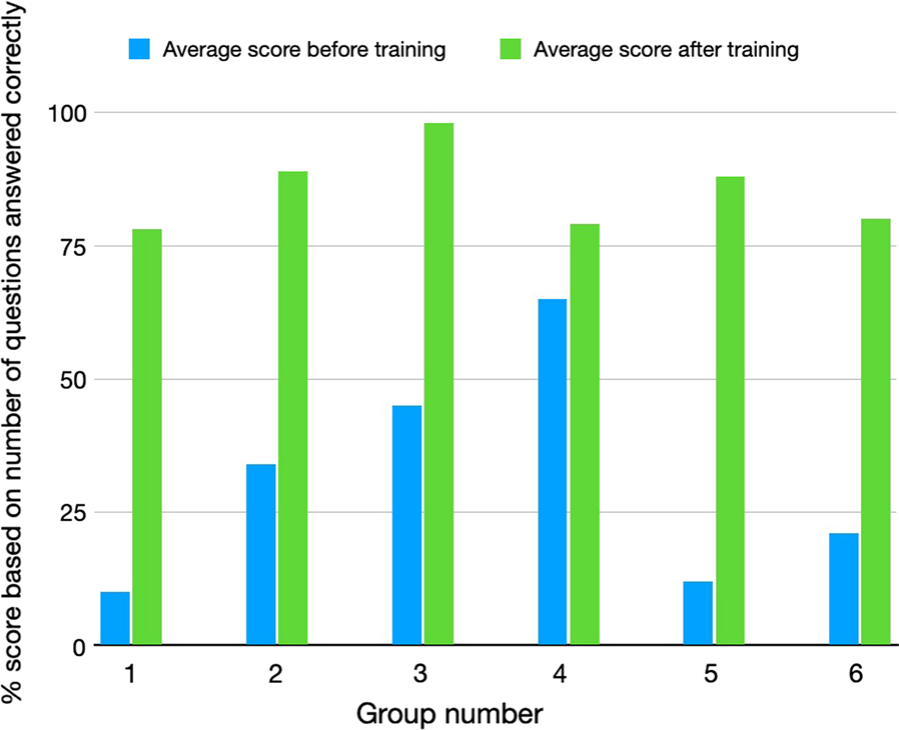
Comparison of the pre-test and post-test scores for 20 questions asked of each participant in each of the 6 different training session groups

A comparison of the pre-training tests with the post training ones was completed, showed a very high degree of learning achieved by virtually all attendees. Figure 3 shows the pre-training and post-training scores obtained by the attendees at each session who completed both the tests. Before training the number of questions answered correctly across the six sessions ranged from an average of 9.4 (with a range between groups of 8.6–10.6 of the 20 questions answered correctly; after training this improved to 16.7 (16.1–20.0). Thus, across all the training sessions there was almost twice as many questions being answered correctly after training, and in each group, there were attendees who got all questions correct or only answered one or two questions incorrectly. The institutional planning projects were all examined and found to be of high quality and to contain all the major needed components for implementation.

### Post training activities

#### MMDP clinics

On returning to their posts after the training session, the participants had discussions with their superintendents and DMOs, organized training program for doctors, nurses and health care workers and carried out various IEC activities. Importantly the State Government allocated funds for the LF MMDP activities in every district as part of the SDG program and now all the districts with clinical LF have started MMDP clinics. Twelve months after the training sessions were completed a total of 82 MMDP clinics which had been started in the state as part of the overall SDG program with an average of 5 facilities per district.

#### Increases in patients receiving care

The training of the medical staff occurred through organized structured sessions over 6 months that included information, hands-on demonstrations and the development of specific LF lymphoedema care plans for the trainees’ home hospitals and medical centers (Fig. 1).

In the following 6 months following the training the number of previously unidentified lymphoedema patients registered and receiving care at these clinics ranged from 296 to almost 400 per clinic, with a total of 3,477 new patients receiving the EPC during this period.

## Discussion

The impairment and disability resulting from the LF-associated lymphoedema, elephantiasis and hydrocele cause a significant public health problem. However, there are relatively simple medical interventions that can address these problems and assist patients. These simple interventions that are central to the training and dissemination of care for those suffering from lymphoedema, and they form the central core of training systems described here. These interventions include treatment for acute attacks (ADL), the reduction in the frequency and severity of ADL with simple hygiene measures, such as washing and basic skin care; which assist in preventing progression of the lymphoedema to the stage of elephantiasis. The currently recommended treatment for bouts of ADL is administration of antibiotics and other supportive measures. The hygiene measures used include: washing the affected parts twice daily with soap and clean water at room temperature and drying carefully with a clean cotton cloth (especially between the toes); maintaining clean nails and treating interdigital lesions (usually with anti-fungal creams); avoiding ‘entry’ lesions through using proper footwear and the use of antiseptic or antibiotic creams to treat small wounds or abrasions. For management of hydrocele, surgical intervention with a hydrocelectomy is the standard option. This is usually a relatively uncomplicated surgical intervention if carried out using adequate surgical procedures and appropriate pre- and post-surgery management.

Prior to the initiation of the GPELF those affected by the lymphedema induced by this parasitic infection, and who suffer the consequent disability and compromised quality of life, have often been unable to obtain the needed care except in a few specialised clinics in concerned countries, such as India, Sri Lanka and Brazil [3, 11–13]. The dissemination of the appropriate care to all patients affected, as the current description of the Kerala Program shows, can occur through well designed and implemented training activities. Success in fulfilling this requirement of the global LF Program by provide care for all those clinically affected with LF is an example of progress towards restoring health equity. This case study demonstrated how equity for a group of people who have, at least until recently, largely been ignored, can be achieved by a set of well designed, simple measures. It is vital that LF patients suffering from lymphoedema obtain the care as defined by GPELF so that these patients have a fair opportunity to reach their full health potential through the provision of care [6]. This requires that the appropriate care they need is available, not only through specialised clinics, but also thorough the national public health system of the endemic area, as has been achieved here in Kerala.

Arguably the two most important factors to the success of this overall venture were, firstly the policy decision (SDG activities) and support of government who saw this as an essential ingredient to eliminate LF and secondly augmenting the medical skills and capability of clinical staff. These two factors have often been difficult to achieve in many endemic countries, although those that have managed to incorporate these components have often achieved success. It is, in general, logically better to follow WHO recommendation to carry out a situational analysis and assessment of local disease LE burden before including specific health facilities in training sessions; however, here in Kerala this was not done, and the inclusion of a particular health facility in training sessions was based on local knowledge of the presence of these patients in the area. It is important to note that the attendees of the training sessions indicated that they had not previously been taking adequate care of LF patients due to a lack of awareness of the required procedures and a lack of facilities. Following the training, the attendees realised that with their newfound knowledge they are now capable of providing quality care to LF patents through simple and affordable measures, which actively improved the quality of life of their patients. It was reported that direct interaction with LF patients and the hands-on activities during training gave them confidence to manage these neglected patients, to become champions for LF care, and to be able to train other health workers in their medical institutions.

The activities presented in this paper, with the training of 184 medical personnel from 82 institutions, are an example of successful integration of healthcare into the public health system in an endemic area. Over 3 years the longstanding WHO Filariasis Center in the Government TD Medical College of Alappuzha, Kerala State, worked with Kerala state government medical officials to develop a solid state wide system of integrating specialised care for LF lymphoedema patients in a public health system, and provided a strong example of achieving equity in healthcare. This is one of first reports of such initiatives in the published literature. It is also a major step towards achieving the goals of GPELF for the people of Kerala, which became the first state in India to achieve this integration of LE care into the public health system, and arguably one of the first amongst all global endemic countries. The approaches taken here are likely to be useful in other countries and Indian states. Kerala’s success in imparting MMDP services to LF patients throughout Kerala is being recognized by the international NTD community as the “Kerala story”.

## Conclusions

This Kerala approach to increasing the provision of support for lymphoedema self-care has demonstrated that generalist health personnel, when appropriately trained, can provide quality lymphoedema care in public health settings, and that patients, when provided services close to their home, are willing to access these services. The key factors in achieving this success in Kerala, which are likely applicable to many situations across the LF endemic world, were: (1) Engaging the support and involvement of senior government officials from the Health and Family Welfare Department. (2) Ensuring the engagement of medical staff from all endemic districts. (3) Participatory training sessions with both active practical and planning components.

This approach, a feasible strategy for integrating long term care for LF patients into a national health system, is an example of moving towards equity in health care for the medically underserved, and successfully addresses an essential target of GPELF.

## Supplementary Information

**Additional file 1:** Objectives and description of various sessions on LF MMDP training in Kerala.

## Data Availability

All data included in this manuscript is available from the primary institute.

## Abbreviations

DMO: District medical officer
IEC: Information, education and communication
LF: Lymphatic filariasis
LE: Lymphoedema
GPELF: Global program for the elimination of lymphatic filariasis
MMDP: Morbidity management and disability prevention
ECP: Essential care package
DMDI: Disease management and disability inclusion
ADL: Adenolymphangitis
NTD: Neglected tropical diseases
SDG: Sustainable development goals
